# Exosomes and mimics as novel delivery platform for cancer therapy

**DOI:** 10.3389/fphar.2022.1001417

**Published:** 2022-10-12

**Authors:** Fuxu Yang, Mingyue Wang, Xingang Guan

**Affiliations:** ^1^ Department of Basic Medicine, School of Medicine, Taizhou University, Taizhou, China; ^2^ Key Laboratory of Pharmaceutics and Bioengineering, School of Medical Technology, Beihua University, Jilin, China; ^3^ Center of Reproductive Medicine and Center of Prenatal Diagnosis, First Hospital, Jilin University, Changchun, China

**Keywords:** exosome, cancer therapy, nanovesicle, drug delivery, tumor targeting

## Abstract

Exosomes are nano-sized biological extracellular vesicles transmitting information between cells and constituting a new intercellular communication mode. Exosomes have many advantages as an ideal drug delivery nanocarrier, including good biocompatibility, permeability, low toxicity, and low immunogenicity. Recently, exosomes have been used to deliver chemotherapeutic agents, natural drugs, nucleic acid drugs, and other antitumor drugs to treat many types of tumors. Due to the limited production of exosomes, synthetic exosome-mimics have been developed as an ideal platform for drug delivery. This review summarizes recent advances in the application of exosomes and exosome-mimics delivering therapeutic drugs in treating cancers.

## 1 Introduction

Malignant tumors are among the leading causes of death worldwide (Mattiuzzi et al., 2019). Current therapeutics, including chemotherapy, radiation, surgical resection, and immunotherapy, remain the most commonly used treatment (Leal and García-Perdomo, 2019). However, many malignant tumors have poor prognoses due to the late diagnosis and lack of effective treatment options. Thus, novel antitumor drugs and treatments are urgently needed to enhance treatment efficacies. Drug delivery systems (DDS) hold great promise in improving cancer treatments (Song et al., 2021). Despite the improved efficacy of reported DDS in treating many types of tumors, high clearance, toxicity to normal tissues, and limited penetrating depth are the main limitations of current nanocarriers for cancer therapy, leading to poor treatment outcomes (Maeda and Khatami, 2018).

Exosomes are nano-sized extracellular vesicles (EVs) ranged from 40 to 100 nm that are secreted by many types of cells (Pegtel and Gould, 2019). Exosomes, apoptotic bodies, and microvesicles are three members of EVs family (Liao et al., 2019). Exosomes, as the smallest EVs, contain various proteins and nucleic acid molecules, essential in transmitting information between cells. Exosomes were first isolated and purified from sheep reticulocytes by Johnstone (Johnstone et al., 1987). Initially, exosomes were thought to be wastes discharged by cells. However, subsequent studies in recent years have shown that exosomes play critical roles in the tumor microenvironment, such as regulating the occurrence and development of tumors (Xiong et al., 2017; Wang et al., 2020). Further investigations have shown that exosomes secreted by tumor cells may have unique properties and could act as biomarkers for tumor diagnosis (Wang et al., 2018). In addition, exosomes exhibited efficient tumor enrichment effects known as high permeability and retention effects (EPR) (Ngoune et al., 2016). Therefore, the appropriate size and unique physiological structure properties of exosomes make them suitable for delivering various reagents for therapeutic applications (Mohammed et al., 2017). In addition, the natural materials-derived biocompatibility, structural stability, good permeability, low toxicity, and immunogenicity make them ideal carriers for drug delivery (Lee et al., 2012; Xin et al., 2012; Lou et al., 2015). Increased studies have indicated the superior effect of drug-loaded exosomes in treating many diseases (Bagheri et al., 2020; Pei et al., 2021).

### 1.1 Origin of exosomes

**FIGURE 1:**
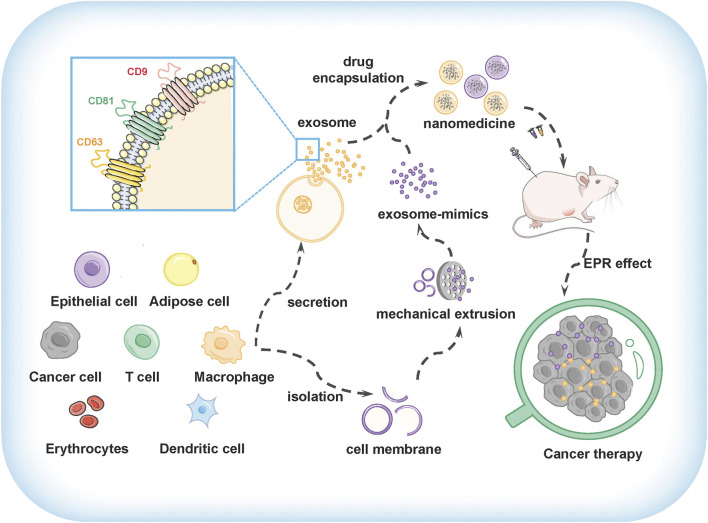
The preparation and application of exosomes and exosome-mimics as drug delivery vehicles for cancer therapy.

Exosomes can be isolated from cell culture supernatants, plasma, serum, and various sources (Muller et al., 2014). The biogenetic processes of exosomes can be divided into four stages: initiation, endocytosis, formation of multivesicular bodies (MVBs), and secretion of exosomes (Figure 1) (Razi and Futter, 2006). The formation of exosomes in MVBs has similarities with lysosomal formation because lysosomal surface proteins such as LAMP and CD63 are also present in exosomes (Johnsen et al., 2014). The production of exosomes is affected by many factors, such as an increase in intracellular Ca^2+^ (Merendino et al., 2010), amino acids, and intracellular and intercellular pH (Fader and Colombo, 2009). Some specific mechanisms have been proposed to explain the various stages of exosome biogenesis, suggesting that exosomal formation may be a fine-tuning process (Van Niel et al., 2018). However, it is still unclear what triggers exosome biogenesis and secretion.

### 1.2 The composition of exosomes

Exosomes from various organisms and cell types contain thousands of proteins, lipids, mRNAs, and miRNAs. (Zhuang et al., 2011). The confluence of cholesterol, diglyceride, phospholipid, sphingolipid, and glycerophospholipid is higher in exosomes than in parental cells (Yang et al., 2015). Specific molecules, including targeting and fusion proteins, cytoplasmic enzymes, chaperones, and membrane transport proteins, are enriched in exosomes (Gonzalez-Begne et al., 2009). CD9, CD63, and CD81 proteins are usually considered marker proteins of exosomes (Subra et al., 2010; Mathieu et al., 2021). Exosomes can transfer their functional molecules from one cell to another *via* cell-to-cell communication (Dourado et al., 2019).

### 1.3 Separation of exosomes

The great potential of exosomes for delivering vehicles prompts the need for high-yield exosomes. Exosomes are usually isolated from cells incubated with serum-free mediums for several days (Gupta et al., 2018; Biadglegne et al., 2021). Although many cells can produce exosomes, the yield of exosomes produced by different cell types is highly variable (Kim et al., 2021). Therefore, selecting the optimal source of cells is crucial for the production of exosomes. Nowadays, the exosomal preparation protocols include differential ultracentrifugation and density gradient centrifugation, sedimentation, capture, and microfluidic separation (Coughlan et al., 2020; Jung et al., 2020). As the most popular protocol, differential ultracentrifugation with a centrifugal force of 200 × g to 100,000 × g removes larger particles and cell debris and finally precipitates exosomes (Thakur et al., 2020).

Due to the unsatisfactory yield, how to increase the production of exosomes is still a significant challenge. Kim *et al.* found that the mesenchymal stem cells (MSCs) cultured in three-dimensional (3D) spheroids produced a higher level of exosome than that in the traditional two-dimensional (2D) culture (Kim et al., 2018). The changes in exosome production may be attributed to the non-adherent and round MSCs, which means the three-dimensional structure may affect the efficiency of exosome production. Ludwig *et al.* found that adenosine receptors modulated the production of exosomes originating from tumor cells (Ludwig et al., 2020). Ambattu *et al.* showed that high-frequency acoustic cell stimulation induced an 8-fold increase in exosome production through irradiation and post-excitation incubation steps (Ambattu et al., 2020). These findings provided new ideas for the preparation of a large number of exosomes. However, obtaining exosomes with high purity and satisfied yield is still a significant problem limiting the application of exosomes in cancer treatment.

## 2 Drug loading into exosomes

Drug loading into exosomes refers to loading different drugs into purified exosomes’ inner cavities or intramembrane. Exosomes could encapsulate hydrophilic drugs, hydrophobic reagents, and membrane proteins as efficient drug delivery vehicles. In principle, drug-loaded exosomes can be acquired through post-loading and pre-loading of exosomes*.*

### 2.1 Post-loading of exosomes

**FIGURE 2:**
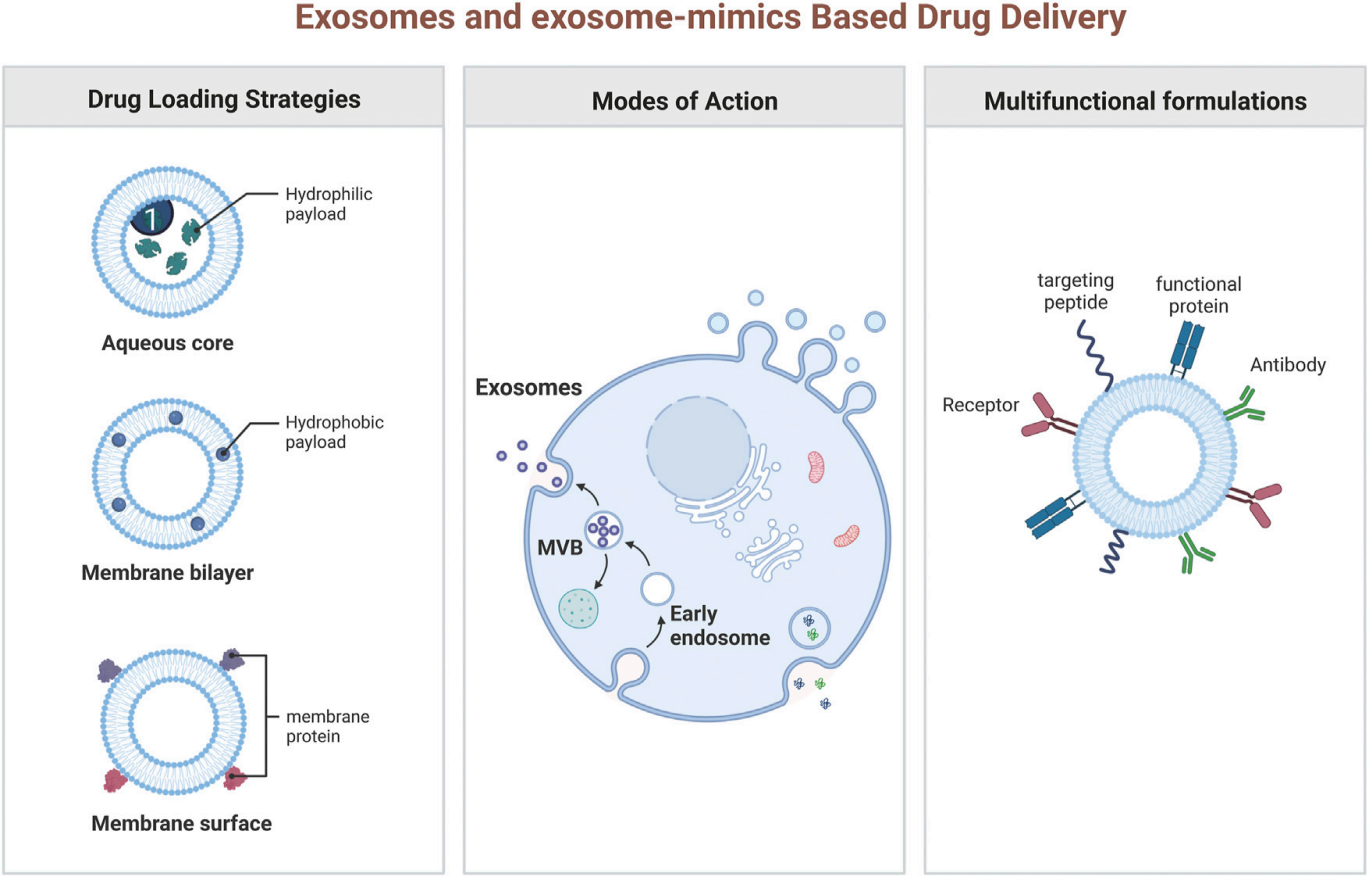
Drug loading strategies of exosomes and exosome-mimics.

There are several ways to load drugs into isolated exosomes, including co-incubation, electroporation, and ultrasound (Değirmenci et al., 2022). Co-incubation is a commonly used method for drug loading into exosomes, which is simple to operate but has low loading efficiency (Fang and Liang, 2021). Yang and his coworkers prepared a linezolid-loaded exosome using co-incubation at 37°C with a drug loading efficiency of 5% (Yang et al., 2018). Nucleic acid drugs, including DNA, siRNA, miRNA, and others were usually loaded into the inner cavity of exosomes by the electroporation method (Figure 2) (Faruqu et al., 2018; Asadirad et al., 2019). The loading efficiency of nucleic acid drugs into exosomes depends on the molecule weight and the size of exosomes (Lamichhane et al., 2015). Unlike the traditional strategies, Yang et al. designed a cell nanoporation (CNP) biochip, combining exosome purification and drug loading into a device, significantly improving the exosome yield and mRNA loading efficiency (Yang et al., 2020). Improving the drug loading capacity of exosomes is crucial for enhancing the efficacy of cancer treatment.

### 2.2 Pre-loading of exosomes

Another drug loading method is introducing the drugs into the exosome–derived cells, which is especially important for those drugs that cannot be loaded into purified exosomes, such as the cytosol and transmembrane proteins (Bai et al., 2020). Tian et al. transfected cells with iRGD plasmid to obtain iRGD-decorated exosomes, encapsulating doxorubicin for targeted therapy in breast cancer (Tian et al., 2014). Severic et al. modified exosome-mimics with prostate-specific targeting peptides, which significantly increased the accumulation of exosome-mimics in prostate tumor tissues and reduced their distribution in normal tissues and organs (Severic et al., 2021).

## 3 Application of exosomes as drug delivery vehicles

### 3.1 Nucleic acid drugs

Recently, exosomes from tumor cells, adipose stem cells, mesenchymal stem cells and epithelial cells have been used to treat different diseases (Zhang M. et al., 2021; Pan et al., 2021; Rui et al., 2021; Sun et al., 2021; Wang L. et al., 2022). Gene therapy has shown great promise in treating intractable diseases (Suresh et al., 2014). The key to gene therapy is introducing nucleic acid drugs into the targeted cells for long-term gene regulation (Misra, 2013). Despite some promising results, the clinical application of gene therapy is limited by the lack of proper delivery systems (Mendell et al., 2021). In this regard, exosomes have been used to deliver many nucleic acid drugs, including miRNA, siRNA, and mRNA (Aqil et al., 2019; Zhang D. et al., 2021; Huang et al., 2021).

#### 3.1.1 miRNA

miRNA is a non-coding RNA molecule with 20–22 nucleotides in size binding to a partially complementary mRNA sequence, resulting in targeted degradation or translational inhibition (Novina and Sharp, 2004). Increasing evidence has shown that the acquisition or loss of related miRNA function is closely related to tumorigenesis (Farazi et al., 2013; Zhou et al., 2014; Ye et al., 2017; Mo et al., 2018). The inherent ability of exosomes in delivering bio-related molecules is a significant advantage over other delivery platforms (Esposito et al., 2021). For example, exosomes modified with GE11 peptide could deliver let-7a miRNA into the epidermal growth factor receptor (EGFR)-overpressed breast cancer cells (HCC70, HCC 1954, and MCF-7). Enhanced tumor suppression was observed in mice treated with miRNA-loaded exosomes in breast cancer (Ohno et al., 2013). Han et al. developed exosomes delivering miR-567 and found that they could target autophagy-related proteins (ATG5) to reverse trastuzumab resistance in breast cancer cells (Han et al., 2020). Yao et al. used exosomes derived from HEK-293T cells to deliver miR-204-5p for cancer treatment. The results showed that exosomal miR-204-5p could significantly inhibit the proliferation of cancer cells and increase their sensitivity to chemotherapeutic agents (Yao et al., 2020).

#### 3.1.2 siRNA

Tumor cells can overcome the immune attack from the host immune system through the immunosuppressive tumor microenvironment (Chew et al., 2012). Cancer immunotherapy results largely depend on the continuous activation and expansion of tumor-specific T cells, especially the tumor-infiltrating cytotoxic T lymphocytes (De Sanctis et al., 2022). Pei et al. used cRGD-modified exosomes to deliver siFGL1 and siTGF-β1, significantly increasing the number of tumor-infiltrating T lymphocytes (Pei et al., 2021). Galectin-9 is a β-galactoside-binding lectin, and its blockade could induce the antitumor immune response (Moar and Tandon, 2021). Galectin-9 was highly expressed in tumor tissues of patients with pancreatic ductal adenocarcinoma (PDAC) (Seifert et al., 2020). Zhou et al. developed exosomes modified with oxaliplatin prodrug to deliver galectin-9 siRNA to PDAC tissues, which induced immunogenic cell death (ICD) of tumor cells and showed significant therapeutic effects on PDAC (Zhou et al., 2021). Shtam et al. prepared exosomes to deliver RAD51-siRNA and promoted the massive reproductive cell death of recipient cancer cells (Shtam et al., 2013). KRAS is a signaling protein that drives pancreatic cancer formation mutations (Diehl et al., 2021). Exosomes delivering siRNA targeting the oncogene protein Kras (KrasG12D) demonstrated unprecedented tumor regression and promising potential for targeting pancreatic cancer (Zorde Khvalevsky et al., 2013; Kamerkar et al., 2017).

#### 3.1.3 mRNA

Many studies have shown that exosomes isolated from many tumor cells contained tumor cell-specific mRNA, and exosomes delivering mRNA has attracted considerable attention for cancer treatment (Gutkin et al., 2016). Mizrak et al. reported that mRNA-loaded exosomes could efficiently deliver mRNA and show a combination therapy effect with other anti-cancer drugs (Mizrak et al., 2013).

### 3.2 Chemotherapy drugs

Chemotherapy remains one of the primary cancer treatments in the clinic. Unfortunately, many chemotherapy drugs are associated with severe adverse events in clinical use (Cassidy and Misset, 2002). Many studies have shown the decreased toxicity of chemotherapeutics -loaded exosomes toward normal tissues for improved cancer therapy (Hadla et al., 2016; Li Y. et al., 2018b). Tian et al. developed iRGD-modified exosomes derived from mouse immature dendritic cells (imDC) to encapsulate doxorubicin for cancer treatment, showing good antitumor efficacy with no toxicity observed (Tian et al., 2014). Wang et al. purified exosomes from M1 type macrophages by ultra-high speed centrifugation and loaded paclitaxel into exosomes by ultrasound in a breast cancer mice model. The paclitaxel-loaded exosomes demonstrated enhanced tumor targeting and inhibited tumor growth compared with free paclitaxel (Wang et al., 2019). Embryonic stem cell-derived exosomes delivering paclitaxel also showed good antitumor efficacy in glioblastoma (Zhu et al., 2019).

Curcumin, a polyphenol enriched in turmeric plants, has a wide range of pharmacological effects, including anti-oxidative stress and inhibition of cell proliferation of malignant tumors. The poor water solubility greatly limited their further applications (Anand et al., 2007). Exosomes derived from cow milk and intestinal epithelial cells could improve the oral bioavailability of curcumin (Carobolante et al., 2020). Moreover, exosomes derived from milk could protect curcumin from metabolism and improve its anti-cancer activity of curcumin (González-Sarrías et al., 2022). Gemcitabine is an effective chemotherapeutic drug for treating pancreatic cancer, but it is often associated with several adverse events in the circulatory system, gastrointestinal tract, and kidneys (Cidon et al., 2018). Li et al. prepared gemcitabine-loaded exosomes, which showed increased tumor accumulation and better tumor inhibition than free gemcitabine (Li et al., 2020). In addition, exosomes have been used to overcome the drug resistance of tumors. Zhang et al. loaded cisplatin into exosomes derived from M1 macrophages of human cord blood (exoCIS) and found that exoCIS could significantly inhibit the growth of cisplatin-resistant ovarian cancer cells (Zhang et al., 2020). Exosome-modified targeting moieties significantly enhanced the antitumor efficacy and reduced the toxicity to normal tissues.

### 3.3 Others

Recently, exosomes have been used to deliver photosensitizers to tumor tissues for cancer therapy (Zhu et al., 2022). Pan et al. loaded PMA/Au-BSA@Ce6 nanoparticles into urinary exosomes and constructed passion fruit-like exosome nanoparticles, which achieved targeted tumor imaging and photodynamic therapy (Pan et al., 2020). Cao et al. prepared vanadium carbide quantum dots-loaded exosomes, which showed a tumor-killing effect through photothermal therapy (Cao et al., 2019). Fan et al. used DNA hinges to connect quantum dots to exosome surfaces (Exosome-DNA-QDs) and found that exosome-DNA-QDs could be phagocytosed by tumor cells faster than normal cells, suggesting the unique advantage of the exosome-based delivery platform for cancer treatment (Fan et al., 2019).

## 4 Exosome-mimics

Exosomes have been an ideal platform to deliver various drugs in treating cancers. However, their further application is limited by the low production yield and lack of targeting properties (Yong et al., 2019; Zhang H. et al., 2021b). Therefore, artificially produced nanovesicles, which mimic the structure of exosomes, have received extensive attention in drug delivery (Nie et al., 2019; Fan et al., 2021; Oroojalian et al., 2021). Exosome-mimics, prepared by extruding whole cells or cell membranes through certain-sized filters, have similar structures and characteristics to exosomes (Li et al., 2022). Synthetic exosome-mimics, retaining the natural properties of cells, can be applied to developing novel therapeutic strategies (He et al., 2021). Some studies have shown that exosome-mimics can result in a 100-fold increase in production yield compared with natural exosomes (Jang et al., 2013).

Until now, exosome-mimics prepared from various cell membranes or platelet membranes could be used in developing cancer vaccination and drug delivery systems (Fang et al., 2014; Zhang et al., 2019). The exosome-mimics encapsulating PLGA nanoparticles showed enhanced stability compared with the bare nanoparticles (Kroll et al., 2017; Zhang et al., 2017). The exosome-mimics prepared by the T cell membrane, which retained T cell receptors on the surface, could bind to HIV surface glycoprotein gp120 and inhibit gp120-induced CD4^+^ T cell damage, suggesting a promising therapeutic agent against HIV infection (Wei et al., 2018b). Exosome-mimics prepared from gastric epithelial cell membranes, macrophage membranes, platelet membranes, and neutrophil membranes have also shown improved efficacies in treating some diseases (Thamphiwatana et al., 2017; Angsantikul et al., 2018; Wei et al., 2018a; Zhang Q. et al., 2018a). Exosome-mimetic nanovesicles have similar abilities as drug delivery systems compared with exosomes (Pisano et al., 2020).

Cancer immunotherapy, which aims to eliminate cancer cells by the host immune system, has attracted significant attention during the past decade (Wang W. et al., 2022b; Peng et al., 2022). Among different cancer immunotherapy strategies, immune checkpoint blockade has shown significant clinical effects in many tumors (Sharpe and Pauken, 2018). Zhang et al. prepared engineered cellular nanovesicles presenting the programmed death-1 (PD-1) receptor, which could selectively bind to programmed death-ligand 1 (PD-L1). The PD-1 nanovesicles induced an antitumor immune response (Zhang X. et al., 2018b). Zha et al. prepared gemcitabine-loaded PD-1 nanovesicles, which significantly suppressed tumor growth in mice, showing the unique advantage of PD-1-decorated exosome-mimics in cancer therapy (Zha et al., 2022). Signal regulatory protein-α (SIRPα) could bind to CD47 molecules on tumors and normal tissues and release a “do not eat me” signal to prevent the phagocytosis of cells (Ring et al., 2017). Rao et al. prepared SIRPα variant-presented extracellular vesicle mimics (SαV-C-NVS), which could disrupt the CD47-SIRPα axis and repolarize TAMs towards the M1 phenotype (Rao et al., 2020). These studies demonstrated the potential of functional exosome-mimics in drug delivery for cancer therapy.

## 5 Conclusion and perspectives

Exosomes and exosome-mimics have shown great potential in delivering various drugs and nanoparticles for cancer therapy. Exosomes derived from dendritic cells (DCs), mesenchymal stem cells (MSCs), and patient tumor cells have been used to deliver tumor antigens or anti-cancer drugs in some clinical trials (Chen et al., 2020). However, many problems remain to be solved to advance the further application of exosomes. For example, the limited yield of exosomes could not satisfy the therapeutic application in preclinical and clinical studies. Exosome-mimics, prepared from cell membranes on a large scale, showed promising potential in treating some diseases. Moreover, the drug loading efficiency into exosomes, especially for nucleic acid drugs, is relatively low. Developing novel strategies with efficient drug loading is urgently needed (Li S.-P. et al., 2018a). In addition, the unsatisfied transfection efficiency of exosomes dramatically influences the final gene regulation results in recipient cells. Further studies are required to improve the exosomal yield and drug loading efficiency. Exosomes and exosome-mimics have promising prospects as ideal drug delivery systems in cancer therapy.
